# Predictive Models of Gas/Particulate Partition Coefficients (*K*_P_) for Polycyclic Aromatic Hydrocarbons and Their Oxygen/Nitrogen Derivatives

**DOI:** 10.3390/molecules27217608

**Published:** 2022-11-06

**Authors:** Qiang Wu, Siqi Cao, Zhenyi Chen, Xiaoxuan Wei, Guangcai Ma, Haiying Yu

**Affiliations:** College of Geography and Environmental Sciences, Zhejiang Normal University, Yingbin Avenue 688, Jinhua 321004, China

**Keywords:** PAHs and oxygen/nitrogen derivatives, gas/particulate partition coefficient (*K*_P_), quantitative structure-activity relationships (QSPR), multiple linear regression (MLR), support vector machine (SVM)

## Abstract

Polycyclic aromatic hydrocarbons (PAHs) and their oxygen/nitrogen derivatives released into the atmosphere can alternate between a gas phase and a particulate phase, further affecting their environmental behavior and fate. The gas/particulate partition coefficient (*K*_P_) is generally used to characterize such partitioning equilibrium. In this study, the correlation between log *K*_P_ of fifty PAH derivatives and their n-octanol/air partition coefficient (log *K*_OA_) was first analyzed, yielding a strong linear correlation (*R*^2^ = 0.801). Then, Gaussian 09 software was used to calculate quantum chemical descriptors of all chemicals at M062X/6-311+G (d,p) level. Both stepwise multiple linear regression (MLR) and support vector machine (SVM) methods were used to develop the quantitative structure-property relationship (QSPR) prediction models of log *K*_P_. They yield better statistical performance (*R*^2^ > 0.847, RMSE < 0.584) than the log *K*_OA_ model. Simulation external validation and cross validation were further used to characterize the fitting performance, predictive ability, and robustness of the models. The mechanism analysis shows intermolecular dispersion interaction and hydrogen bonding as the main factors to dominate the distribution of PAH derivatives between the gas phase and particulate phase. The developed models can be used to predict log *K*_P_ values of other PAH derivatives in the application domain, providing basic data for their ecological risk assessment.

## 1. Introduction

Polycyclic aromatic hydrocarbons (PAHs) are typical persistent organic pollutants (POPs) that are widely found in the environment [1]. Exposure to PAHs may lead to atherosclerosis, hypertension and myocardial infarction, and increase the risk of skin, lung, pancreas, stomach, intestinal and other cancers [2,3,4,5,6]. PAHs can further undergo photochemical reactions or be oxidized by atmospheric oxidants, such as O_3_, OH radicals and NO_x_, to generate oxygen/nitrogen derivatives, including oxidized PAHs (O-PAHs), nitro PAHs (N-PAHs) and azaarenes (AZAs) [7,8,9]. In addition, the incomplete combustion of fuels during human activities, vehicle and ship exhaust emissions, and industrial waste emissions also lead to the generation of PAHs and their oxygen/nitrogen derivatives [10,11,12,13]. In recent years, PAHs and their oxygen/nitrogen derivatives have been detected not only in the atmosphere, but also in soil, water, sediment and other environmental media and organisms [14,15,16,17,18]. Minero et al. [19] detected N-PAHs in atmospheric particles of Antarctica in the year 2010, indicating the global presence of PAH derivatives. PAH derivatives are generally trace compounds with concentrations of about one-tenth or even one-hundredth of the parent level in environmental media [20,21]. However, most PAH derivatives are direct mutagens or potential carcinogens [22], and some N-PAHs are even 10 times more carcinogenic or 100,000 times more mutagenic than their parent compounds [23,24,25], bringing high risk to human health and arousing widespread concern.

When PAHs and oxygen/nitrogen derivatives are released into the atmosphere from various sources, they can partly exist in gaseous form or partly combine with atmospheric particles to migrate over long distances, and finally move to the ground surface through atmospheric deposition [26]. Studying the distribution of these compounds between the atmosphere and particulate matter has great implications for understanding their environmental behavior and fate. The gas/particulate partition coefficient, *K*_P_, is often used to characterize such distribution equilibrium of organic pollutants and calculated by [27].

(1) $$K_{P}=\frac{C_{P}}{C_{A}\;\times \;\mathrm{TSP}}$$

Here, *C*_P_ represents the concentration of organic matter in the atmospheric particle phase and *C*_A_ represents the concentration in the air phase, with the unit of ng/m^3^; TSP refers to the concentration of total suspended particulate matter, in μg/m^3^.

Determining the *K*_P_ values is time-consuming, laborious, and limited by standard samples of target compounds. Therefore, establishing a predictive model for *K*_P_ can provide an important method to study the gas/particulate distribution behavior of pollutants and supply basic data for their ecological environment safety and health risk assessment.

Previous studies have shown that the n-octanol/air partition coefficient (*K*_OA_) can predict the *K*_P_ values of organic pollutants such as PAHs, polychlorinated biphenyls (PCBs), polychlorinated naphthalenes (PCNs), and DDT [28,29]. However, the low prediction accuracy and the lack of *K*_OA_ values for some compounds restricts the application of this method. A quantitative structure-property relationship (QSPR) model can be used to establish a quantitative relationship between compound properties, environmental behavior parameters and molecular structure feature through mathematical methods, and can further predict the properties and environmental behavior of similar compounds which lack experimental data [30,31,32,33,34].

Therefore, the goals of this study are first to analyze the correlation between log *K*_P_ and log *K*_OA_ of PAHs and oxygen/nitrogen derivatives, and then establish a QSPR prediction model for log *K*_P_ by multiple linear regression (MLR) and support vector machine (SVM) methods. The model performance will be validated and evaluated, and the relevant mechanism and application domain will be discussed to further understand the partitioning process and predict more chemicals.

## 2. Materials and Methods

### 2.1. Log K_P_ Experimental Values

In this study, the experimental *C*_A_, *C*_P_ and TSP values of 50 PAHs and oxygen/nitrogen derivatives were obtained from the previous study [35], including 22 parent and alkyl PAHs, 15 O-PAHs, 9 N-PAHs and 4 AZAs. Then, the log *K*_P_ value for every chemical is calculated by Equation (1). Information about all compounds as well as log *K*_P_ data are listed in Table 1.

### 2.2. Descriptors

The intermolecular interactions, such as van der Waals forces (e.g., dispersion, dipole-dipole, dipole-induced dipole Interactions) and specific polarization (e.g., hydrogen bonding), are important factors to determine the distribution of organic chemicals between gas and particulate phases [36,37]. In this study, the molecular volume (*V*, cm^3^/mol), the dipole moment (*d*, Debye), the square of dipole moment (*d*^2^, Debye) and the average molecular polarization (*α*, a.u.) are selected to characterize dispersion, dipole–dipole and dipole-induced dipole interactions. The frontier molecular orbitals (*E*_LUMO_ and *E*_HOMO_, eV), hardness (*η*, eV), softness (*σ*, eV), chemical potential (*μ*, eV) and electrophilic index (*ω*, eV) are used to quantify the ability of molecules to receive or provide electrons. Three charge descriptors, the most positive electrostatic charge of hydrogen atom (*q*H^+^, a.c.u.), the most positive electrostatic charge of carbon atom (*q*C^+^, a.c.u.) and the most negative electrostatic charge of carbon atom (*q*C^−^, a.c.u.), are employed to characterize the charge information of the compounds. All 13 descriptors were obtained from the output files of molecular configuration optimization, which was carried out by using Gaussian 09 software [38] at M062X/6-311 + G (d, P) level. In addition, the parameters that characterize the electrostatic potential on the molecular surface are selected, including the most positive/negative electrostatic potential on the molecular surface (*V*_s.max_/*V*_s.min_, eV), the average value of the positive/negative electrostatic potential on the molecular surface (${\bar{V}}_{s}^{+}$/${\bar{V}}_{s}^{-}$, eV), the average dispersion of the electrostatic potential on the molecular surface (*Π*, eV), and the equilibrium constant of the electrostatic potential on the molecular surface (*τ*). These parameters were further calculated by GsGrid (Verison 1.7) software [39] based on the Gaussian output files. Moreover, the log *K*_OA_ values of the compounds were calculated using EPI Suite^TM^ v4.11 software [40].

### 2.3. Model Construction and Verification

The stepwise regression method in IBM SPSS 21.0 software [41] was used to screen variables and build the MLR model. The regression performance, predictive capability and robustness of the model was assessed according to the OECD guidelines [42], the square of the correlation coefficient (*R*^2^), the square of the prediction correlation coefficient (*Q*^2^), the root-mean-square error (*RMSE*), the mean absolute error (*MAE*), the maximum positive error (*MPE*), the maximum negative error (*MNE*), and the systematic error (*BIAS*) were calculated to evaluate the fitting ability of the model. Then the original data set was randomly divided into a training set (70%) and a test set (30%) for simulated external authentication to evaluate the predictive ability. The leave-one-out cross-validation was further implemented by Weka 3.8.0 software [43], and the mean cross-validation correlation coefficient (*Q^2^*_CV_) and the mean root-mean-square error (*RMSE*_CV_) were obtained to evaluate the robustness of the QSPR model.

In order to further improve the model’s performance, an SVM model was constructed using R language by the descriptors employed in the MLR model. The kernel function of the SVM method is radial basis function. The complexity and prediction error of the model were determined by searching for the optimal combination of hyperparameters (γ and C), and the optimal model is obtained based on it. In this process, the range of the combination of *γ* and *C* was set as 10^−2^~10^4^, and the grid search method was used to find the optimal combination. The ten-fold cross validation method was used to evaluate the performance of the SVM model. At the same time, the counter map of log γ and log C was drawn to visualize the combination of the hyperparameters.

### 2.4. Define the Application Domain

The model application domain was defined using a Williams diagram [33]. If the absolute value of the standardized residual (*StdR*) of a compound is smaller than 3, it is considered to be well predicted. If the leverage value *h_i_* of a compound is larger than the threshold h^*^ (h^*^ = 3 × *p*/*n*, *p* represents the number of molecular structure descriptors, *n* represents the number of modeling data), this compound may have extreme descriptors that can influence the model construction, so it is identified as a high-influence compound. It should be noted that if the absolute values of *StdR* of the high-influence compounds are less than 3, this indicates the model has great generalization capability.

## 3. Results and Discussion

### 3.1. Model Establishment and Verification

(1) log *K*_OA_ model

The linear correlation between log *K*_P_ and log *K*_OA_ was obtained:

log *K*_P_ = (0.643 ± 0.046) × log *K*_OA_ + (−8.287 ± 0.410) (2)

**Figure 1:**
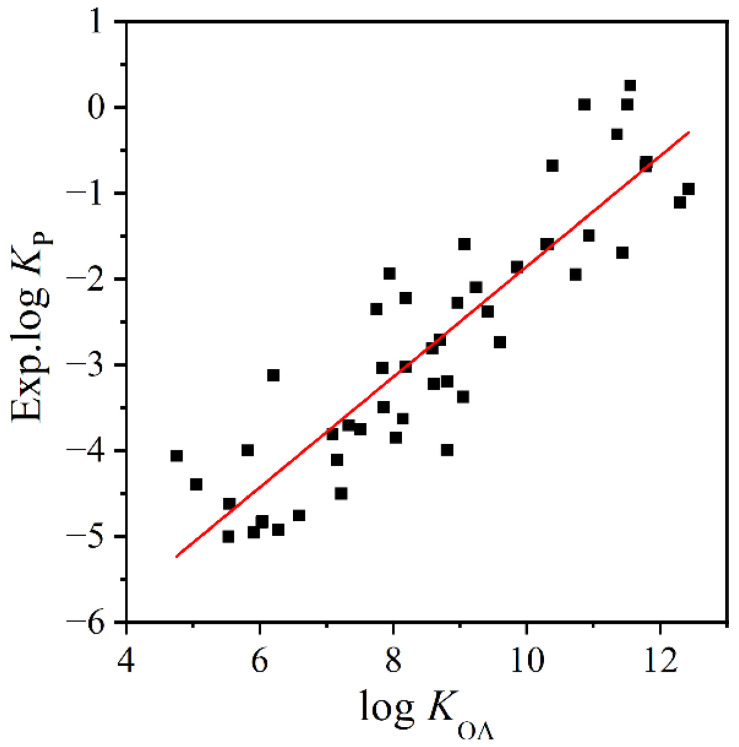
The fitting plot between experimental log *K*_P_ values and log *K*_OA_ values.

As shown in Figure 1, log *K*_P_ positively correlates with log *K*_OA_ (*p* < 0.05). This indicates log *K*_OA_ can be used to roughly predict log *K*_P_ values. The predictive values are listed in Table 1 and the 95% confidence intervals are provided in Supplementary Materials, Table S1. However, the moderate correlation coefficient (*R*^2^ = 0.801) may lead to inaccurate predictive results. QSPR models are further developed.

(2) MLR model

The QSPR model was established by stepwise MLR method based on quantum chemical descriptors:

log *K*_P_ = (0.031 ± 0.002) × *α* + (−24.453 ± 3.684) × *V*_s.min_ + (−9.358 ± 0.433) (3)

This model has two molecular descriptors *α* and *V*_s.min_, both of which have *VIF* values less than 10 (see Table 2), and there is no multicollinearity in the model (*p* < 0.05). The predictive log *K*_P_ values as well as the calculated values of the employed descriptors are shown in Table 1, and the 95% confidence interval of the predictive log *K*_P_ values can be found in Table S1.

The statistical performance of MLR model based on quantum chemical descriptors has been significantly improved: *R*^2^ = 0.847, *Q*^2^ = 0.847, and *RMSE* = 0.584 (Table 3), indicating the model has a good fitting performance. It can be seen from Table 3 that the training set (70%) and validation set (30%) of simulated external validation have similar statistical parameters with the MLR model: *R*^2^ = 0.842, *Q*^2^ = 0.842, and *RMSE* = 0.618 (training set); *R*^2^ = 0.854, *Q*^2^ = 0.847, and *RMSE* = 0.535 (validation set); *R*^2^ = 0.847, *Q*^2^ = 0.847, *RMSE* = 0.584 (MLR model based on whole dataset). The regression coefficients of the descriptors in the model established by the training set are also close to those of the MLR model, 0.031 for *α* in both models based on training set and the whole dataset; −27.835 and −24.453 for *V*_s.min_ for training set and the whole dataset, respectively. Moreover, Roy et al. [44,45] have pointed out a serial criterion to detect the existence of systematic error and to judge the predictive ability. We also applied this criterion to our validation set, and the calculation results were: (1) the ratio of number of positive and negative errors NPE/NNE = 1.143, no larger than 5; the absolute value of mean positive error / mean negative error ABS (MPE/MNE) = 0.903, smaller than 2; the difference between the average absolute error (MAE = 0.438) and absolute of average value (ABS (BIAS) = 0.002) is 0.436, larger than 0.5 × MAE; *R*^2^ (*i*th vs (*i* − 1)th residuals) = 0.099, smaller than 0.5; *R*^2^ (log *K*_P_ vs. residuals) = 0.029, smaller than 0.5; (2) after removing the two highest residual values (5%), the MAE (0.370) is smaller than 0.1 × log *K*_P_ range of training set (5.21) and MAE + 3σ (standard deviation of the absolute error, 0.234) is very close to 0.2 × log *K*_P_ range of training set. These results show that the developed MLR model has no systematic error and good predictive ability. In the leave-one-out cross-validation, the average *Q*^2^_CV_ and *RMSE*_CV_ is 0.906 and 0.625, respectively, which further proves the robustness of the developed MLR model [46].

**Figure 2:**
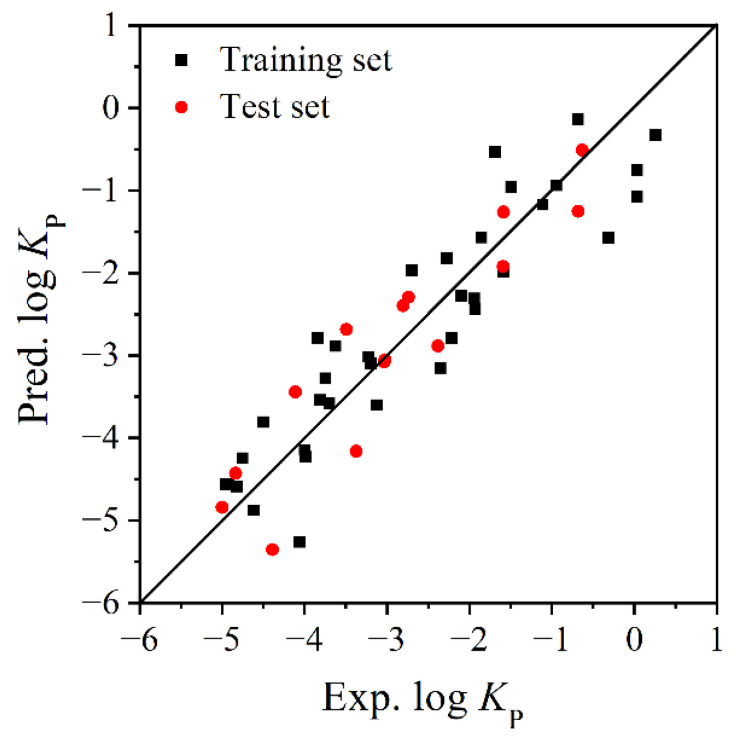
Fitting plot of experimental and predictive log *K*_P_ values by MLR model.

**Figure 3:**
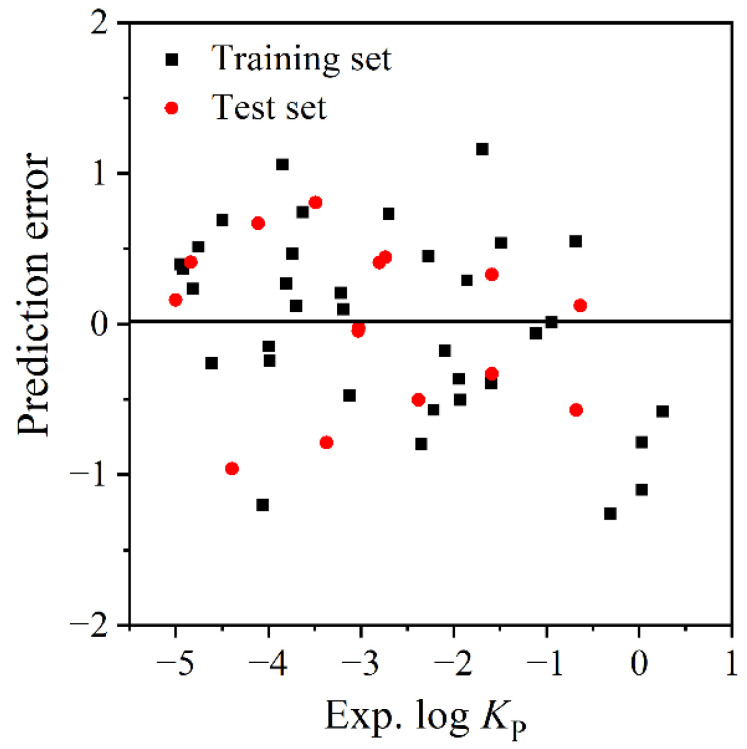
The distribution of predictive log *K*_P_ errors by MLR model.

The fitting plot of the experimental log *K*_P_ values and the predicted log *K*_P_ values by the MLR model (Figure 2) shows they have great agreement. Figure 3 shows that the predictive errors of log *K*_P_ are randomly distributed, and they have no dependence on the experimental value. This conclusion can also be verified by the BIAS = 0.000 of the MLR model (Table 3).

(3) SVM model

**Figure 4:**
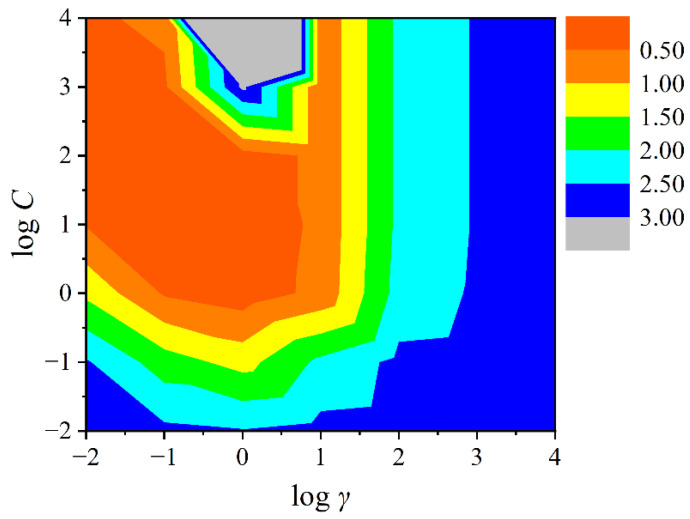
Contour map of the combination of hyperparameter (*γ*) and penalty factor (*C*) in the SVM model.

In order to check whether the machine learning method could improve the statistical performance of the model, SVM model is further established basing on the descriptors (*α* and *V*_s.min_) that are screened by MLR. The contour map of the combination of hyperparameter *γ* and penalty factors *C* is shown in Figure 4. It shows that the smallest predictive errors of the model (<0.50) exist in the brown area, and the largest predictive errors appear in the gray area. The optimal combination is *γ* = 0.1, *C* = 10, which yields the following evaluation parameters (*N* represents the number of data points in the data set):

*N* = 35, *R*^2^ = 0.908, *RMSE* = 0.465, *Q*^2^ = 0.853 (training set)

*N* = 15, *R*^2^ = 0.813, *RMSE* = 0.572, *Q*^2^ = 0.818 (validation set)

**Figure 5:**
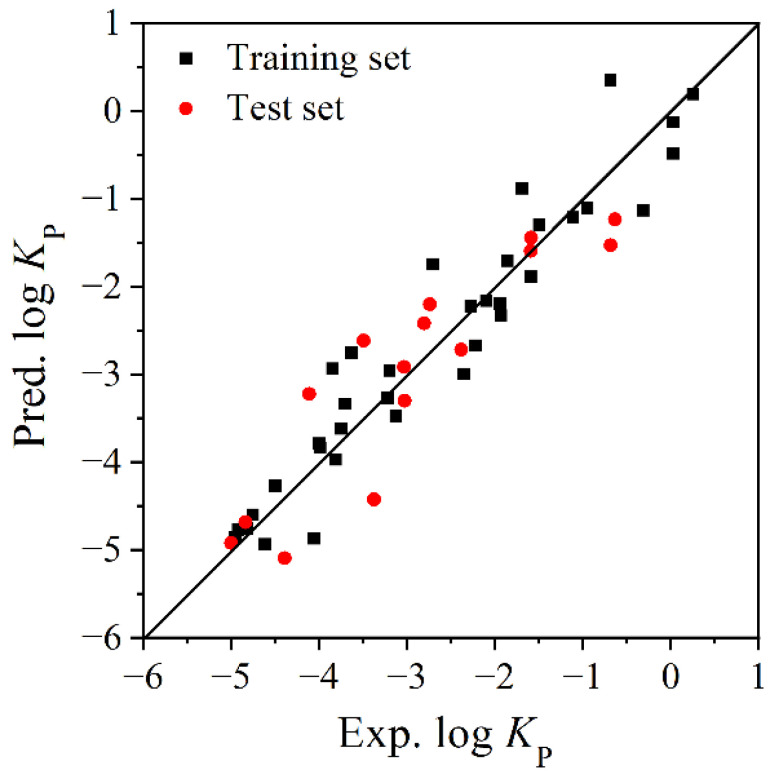
Fitting plot of experimental and predictive log *K*_P_ values by SVM model.

The model also has good fitting ability and robustness, as shown by the high *R*^2^ (0.908) and *Q*^2^ (0.853) values. In external validation, both *R*^2^ (0.813) and *Q*^2^ (0.818) values are greater than 0.8, further indicating a good predictive ability. Figure 5 also shows a good agreement between the experimental log *K*_P_ values and the predictive values calculated by the SVM model.

(4) Comparison of the different models

The *R*^2^ values of the log *K*_OA_ model, the MLR model and the SVM model are all greater than 0.8, indicating every model has good fitting ability. In comparison, the MLR model has better performance than the log *K*_OA_ model. The training set of SVM model obtains the highest *R*^2^ value among the three models; however, the *R*^2^ of its validation set is relatively lower than that of MLR model. As a black-box model, the prediction of the SVM model is an opaque process which cannot provide more information, such as the relationship between the molecular descriptors and the target endpoint under study, thus limiting its application. Furthermore, the MLR model based on molecular structure descriptors avoids the difficulties of experimental measurement, and is a visual model, making it simpler and more convenient for practical application. According to the comprehensive comparison, the MLR model is considered as the optimal predictive log *K*_P_ model for the following analysis.

### 3.2. Characterization of the Model Application Domain

**Figure 6:**
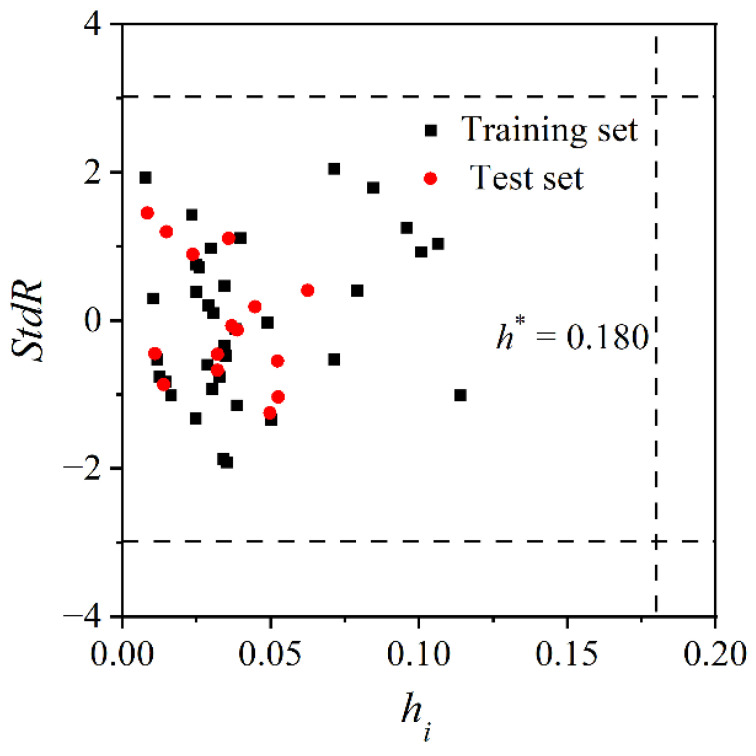
Williams diagram of MLR model.

Figure 6 shows the Williams diagram of the MLR model with threshold h^*^ = 0.180. All data points locate at the left of h^*^, and the absolute values of *StdR* for all compounds are less than 3, indicating the accurate predictive of this model. Therefore, the MLR model has good applicability and can be used to predict the log *K*_P_ values of compounds in the descriptor domain (*α*: 99.753~280.623; *V*_s.min_: −9.535 × 10^−2^~−2.498 × 10^−2^).

### 3.3. Mechanism Analysis

The MLR model contains two descriptors, the average molecular polarization α and the most negative electrostatic potential on the molecular surface *V*_s.min_. *α* has the highest correlation with log *K*_P_ with the correlation coefficient *R* of 0.839, indicating the great importance of average molecular polarizability in affecting the distribution of PAHs and oxygen/nitrogen derivatives between gas phase and atmospheric particle phase. *α* characterizes the dispersive interaction between the molecules, and a larger α corresponds to a stronger intermolecular dispersive effect [47,48]. Because of the great distance between molecules in the air, the dispersion interaction mainly occurs between atmospheric particles and chemical molecules. Therefore, a larger α leads to stronger dispersion interactions between chemical molecules and particles, and further results in a larger log *K*_P_. Thus, *α* yields a positive coefficient (0.031) in the model. The second descriptor, *V*_s.min_, shows negative correlation with log *K*_P_ (the coefficient of −24.453). *V*_s.min_ reflects the contribution of molecular electrostatic hydrogen bonds; that is, it reflects the ability of molecules to accept protons to form hydrogen bonds. The smaller *V*_s.min_ value indicates a higher electron density and a stronger ability to accept protons to form hydrogen bonds [49,50]. Therefore, PAHs and oxygen/nitrogen derivatives with smaller *V*_s.min_ values are more likely to combine with atmospheric particulates which have complex compositions.

### 3.4. Discussion

Yuan et al. [51] constructed a temperature-dependent QSPR model for predicting the log *K*_P_ values of 10 PAHs compounds based on molecular structure descriptors and ambient temperature (T). The model included also the descriptor *α* as well as variable T; however, its statistical performance is not satisfactory: *R*^2^ = 0.624, *Q*^2^ = 0.624, and *RMSE* = 0.395. Sun et al. [52] established a Theoretical Linear Solution Energy Relationship (TLSER) model for some organic compounds, including alkanes, alkalic acids, PAHs, O-PAHs and N-PAHs (Table 4), in which *K*_P1_ and *K*_P2_ represent *K*_P_ values measured by 190 m^3^ and 25 m^3^ smoke chambers, respectively. These models show that dispersion and hydrogen bonding are important factors affecting *K*_P_ values, which is consistent with the results of this study. However, the TLSER models contain fewer PAH, O-PAH and N-PAH data. Furthermore, the number of descriptors used in this study is less, which makes it easier to apply.

## 4. Conclusions

In this study, the correlation between the log *K*_P_ and log *K*_OA_ of PAHs and their oxygen/nitrogen derivatives is first analyzed, and then QSPR models for log *K*_P_ prediction are constructed based on quantum chemical descriptors by MLR and SVM algorithms. The QSPR models have better fitting performance, predictive ability and robustness. The mechanism analysis shows that the major factors affecting the distribution of PAHs, O-PAHs, N-PAHs and AZA in the gas and particle phases are intermolecular dispersion and hydrogen bonding. Although the SVM model is slightly superior to the MLR model, it is a black-box model with poor transparency and is dependent on the descriptor screening of MLR process, limiting its further application. In contrast, the MLR model has simple and visualized mathematical expression, bringing convenience to the analysis of the important factors that affect the partitioning of these chemicals between gas and atmospheric particulate phases according to the chemical information carried by the quantum chemical descriptors. Thus, the MLR model can be used to predict the log *K*_P_ values of other PAHs and oxygen/nitrogen derivatives, with the average molecular polarization within 280.623 and 99.753 and the most negative electrostatic potential on the molecular surface *V*_s.min_ within −2.498 and −9.535. The log *K*_P_ values can provide basic data for their environmental fate and ecological risk assessment.

## Figures and Tables

**Table 1:** Experimental and predicted log *K*_P_ values, log *K*_OA_ values, and molecular structure descriptors employed in the QSAR model for 50 PAHs and their oxygen/nitrogen derivatives ^a^.

Compound	Abbreviations	log *K*_P_				log *K*_OA_	*α*	*V*_S.min_ (×10^−2^)
		Exp.	Pred. (log *K*_OA_)	Pred. (MLR Model)	Pred. (SVM Model)			
1,2,3,4-Tetrahydronaphthalene	TH-NAPH	−4.060	−5.231	−5.184	−4.867	4.75	108.571	−3.397
Naphthalene	NAPH ^b^	−4.392	−5.038	−5.239	−5.093	5.05	112.345	−2.698
2-Methylnaphthalene	2-MNAPH ^b^	−5.001	−4.729	−4.738	−4.920	5.53	126.847	−2.924
1-Methylnaphthalene	1-MNAPH	−4.617	−4.716	−4.789	−4.932	5.55	125.047	−2.944
Biphenyl	BIPH	−4.955	−4.484	−4.469	−4.851	5.91	137.036	−2.739
1,3-Dimethylnaphthalene	1,3DMNAPH ^b^	−4.837	−4.407	−4.330	−4.680	6.03	139.231	−3.030
Acenaphthylene	ACEY	−4.921	−4.253	−4.476	−4.766	6.27	134.493	−3.034
Acenaphthene	ACEN	−4.821	−4.401	−4.511	−4.750	6.04	132.491	−3.141
Fluorene	FLUO	−4.756	−4.047	−4.163	−4.599	6.59	145.606	−2.912
Phenanthrene	PHE	−4.500	−3.642	−3.724	−4.268	7.22	162.006	−2.643
Anthracene	ANT	−3.811	−3.725	−3.459	−3.967	7.09	170.616	−2.639
2-Methylphenanthrene	2-MPHE	−3.747	−3.461	−3.205	−3.614	7.50	177.433	−2.820
3,6-Dimethylphenanthrene	3,6-DMPHE	−3.847	−3.120	−2.728	−2.930	8.03	191.260	−3.031
Fluoranthene	FLUA	−3.223	−2.754	−2.946	−3.266	8.60	186.008	−2.796
Pyrene	PYR ^b^	−3.027	−3.017	−2.950	−3.300	8.19	187.779	−2.555
Retene	RET	−2.703	−2.689	−1.919	−1.743	8.70	217.138	−3.080
Benzo[a]anthracene	BaA ^b^	−1.592	−2.451	−1.828	−1.593	9.07	223.989	−2.590
Benzo[e]pyrene	BeP	−0.316	−0.984	−1.513	−1.130	11.35	234.532	−2.550
Benzo[a]pyrene	BaP	0.028	−1.300	−1.016	−0.482	10.86	250.507	−2.568
Indeno [1,2,3-cd]pyrene	IcdP	0.255	−0.856	−0.284	0.192	11.55	272.695	−2.774
Dibenzo[a,h]anthracene	DahA	−0.687	−0.708	−0.094	0.352	11.78	280.623	−2.553
Benzo[g,h,i]perylene	BghiP	0.028	−0.888	−0.702	−0.127	11.50	261.269	−2.498
1-Indanone	1-IND	−3.998	−4.542	−4.235	−3.784	5.82	99.753	−8.388
1,4-Naphthoquinone	1,4-NQ	−3.990	−2.625	−4.261	−3.834	8.80	113.590	−6.535
1-Naphthaldehyde	1-NALD ^b^	−4.111	−3.680	−3.506	−3.224	7.16	127.809	−7.844
2-Biphenylcarboxaldehyde	2-BPCA ^b^	−3.491	−3.236	−2.760	−2.615	7.85	149.944	−8.101
9-Fluorenone	9-FLU	−3.630	−3.050	−2.959	−2.748	8.14	148.889	−7.418
1,2-Acenaphthenequinone	1,2-ACEQ	−3.196	−2.625	−3.180	−2.953	8.80	138.303	−7.854
9,10-Anthraquinone	9,10-AQ ^b^	−2.382	−2.233	−2.902	−2.718	9.41	159.881	−6.271
1,8-Naphtalic anhydride	1,8-NA ^b^	−3.033	−3.243	−3.140	−2.912	7.84	141.118	−7.659
4H-Cyclopenta[d,e,f]phenanthrenone	4-CPHE ^b^	−2.739	−2.110	−2.345	−2.201	9.60	170.679	−7.191
2-Meth-9,10-anthraquinone	2-MAQ	−1.944	−1.383	−2.362	−2.194	10.73	175.048	−6.566
Benzo[a]florenone	BAFLU ^b^	−1.590	−1.660	−1.322	−1.442	10.30	203.092	−7.291
7H-Benzo[d,e]anthracene-7-one	BdeAQ ^b^	−0.682	−1.608	−1.328	−1.527	10.38	199.470	−7.715
Benzo[a]anthracene-7,12-dione	BaAQ	−1.112	−0.373	−1.231	−1.211	12.30	214.077	−6.284
5,12-Naphthacenequinone	5,12-NQ	−0.949	−0.296	−1.006	−1.105	12.42	219.462	−6.523
6H-Benzo[c,d]pyren-6-one	BcdPQ ^b^	−0.635	−0.701	−0.592	−1.231	11.79	222.901	−7.780
1-Nitronaphthalene	1-NNAP	−3.703	−3.571	−3.635	−3.333	7.33	129.792	−7.060
2-Nitrobiphenyl	2-NBP	−2.352	−3.301	−3.184	−2.993	7.75	151.356	−6.187
5-Nitroacenaphthene	5-NACE	−2.219	−3.017	−2.867	−2.671	8.19	151.022	−7.526
2-Nitrofluorene	2-NFLU	−1.932	−3.178	−2.501	−2.329	7.94	167.360	−6.969
9-Nitrophenanthrene	9-NPHE	−2.098	−2.342	−2.324	−2.158	9.24	177.214	−6.454
9-Nitroanthracene	9-NANT	−1.858	−1.943	−1.660	−1.703	9.86	190.063	−7.545
1-Nitropyrene	1-NPYR	−1.496	−1.255	−1.048	−1.295	10.93	211.741	−7.317
2,7-Dinitrofluorene	2,7-DNFLU	−1.595	−1.647	−2.037	−1.888	10.32	187.649	−6.309
6-Nitrochrysene	6-NCHR	−1.696	−0.933	−0.604	−0.879	11.43	232.917	−6.475
Quinoline	QUI	−3.127	−4.298	−3.731	−3.475	6.20	107.069	−9.535
Benzo[h]quinoline	BhQ ^b^	−2.804	−2.767	−2.483	−2.417	8.58	156.877	−8.358
Acridine	ACR	−2.275	−2.522	−1.969	−2.222	8.96	165.008	−9.437
Carbazole	CAR ^b^	−3.372	−2.471	−4.071	−4.423	9.04	145.738	−3.265

^a^ *α* (a.u.) represents the average molecular polarizability; *V*_s.min_ (eV) represents the most negative electrostatic potential on the molecular surface; ^b^ as the validation set in simulated external validation.

**Table 2:** The coefficients, *t*-test (*t* value), significance level (*p* value) and variance inflation factor (*VIF* value) of each descriptor in the MLR model.

Parameter	Coefficient	*t*	*p*	*VIF*
*α*	0.031	15.839	<0.001	1.056
*V*_s.min_	−24.453	−6.638	<0.001	1.056

**Table 3:** Statistical parameters of the MLR model and the simulated external validation.

	*N*	*R* ^2^	*Q* ^2^	*RMSE*	*BIAS*	*MAE*	*MPE*	*MNE*
MLR model	50	0.847	0.847	0.584	0.000	0.491	1.119	−1.197
Training set	35	0.842	0.842	0.618	0.000	0.509	1.162	−1.259
Validation set	15	0.854	0.847	0.535	0.002	0.438	0.807	−0.961

**Table 4:** Comparison of literature models.

Compound	Model	Characterization Results	References
PAHs	log *K*_P_ = (0.018 ± 0.003) × *α* + (−0.080 ± 0.033) × *T* + (18.245 ± 9.979)	*N* = 28, *R*^2^ = 0.624, *Q*^2^ = 0.624, *RMSE* = 0.395	[45]
Organic chemicals	log (10^3^ *K*_P1_) = −17.426 + 0.406 × *d* + 0.058 × *α* − 0.580 × *E*_HOMO_ + 10.236 × *q*H^+^	*N* = 15, *R*^2^ = 0.971, *Q*^2^ = 0.971, *RMSE* = 0.185	[46]
	log (10^3^ *K*_P2_) = −21.307 + 0.162 × *d* + 0.0424 × *α* − 1.531 × *E*_HOMO_ − 0.582 × *E*_LUMO_	*N* = 17, *R*^2^ = 0.839, *Q*^2^ = 0.839, *RMSE* = 0.634	
PAHs, O-PAHs, N-PAHs	log *K*_P_ = (0.031 ± 0.002) × *α* + (−24.453 ± 3.684) × *V*_s.min_ + (−9.358 ± 0.433)	*N* = 50, *R*^2^ = 0.847, *Q*^2^ = 0.847, *RMSE* = 0.584	This research

## Supplementary Materials

The following supporting information can be downloaded at: https://www.mdpi.com/article/10.3390/molecules27217608/s1, Table S1: The lower and upper limit of 95% confidence interval of log KOA model, MLR model and SVM model.
