# Supplementary material for: Predictive Models of Gas/Particulate Partition Coefficients (KP) for Polycyclic Aromatic Hydrocarbons and Their Oxygen/Nitrogen Derivatives
Source: Molecules. 2022 Nov 6;27(21):7608. doi: 10.3390/molecules27217608 (PMC9657024; doi:10.3390/molecules27217608)
Supplement: Supplementary file 1 [file molecules-27-07608-s001.zip › molecules-1959553-supplementary.pdf]

Table S1. The lower and upper limit of 95% confidence interval of log K_{OA} model, MLR model and SVM model.

Compound	Abbreviations	Exp. log K_P	Log K_{OA} model			MLR prediction		
			Pred. log K_P	LLCI	ULCI	Pred. log K_P	LLCI	ULCI
1,2,3,4-Tetrahydronaphthalene	TH-NAPH	−4.060	−6.644	−3.817	−6.644	−5.184	−6.436	−3.932
Naphthalene	NAPH[b]	−4.392	−6.444	−3.631	−6.444	−5.239	−6.496	−3.981
2-Methylnaphthalene	2-MNAPH[b]	−5.001	−6.126	−3.332	−6.126	−4.738	−5.980	−3.494
1-Methylnaphthalene	1-MNAPH	−4.617	−6.112	−3.320	−6.112	−4.789	−6.032	−3.543
Biphenyl	BIPH	−4.955	−5.874	−3.095	−5.874	−4.469	−5.708	−3.229
1,3-Dimethylnaphthalene	1,3DMNAPH[b]	−4.837	−5.795	−3.019	−5.795	−4.330	−5.564	−3.095
Acenaphthylene	ACEY	−4.921	−5.637	−2.869	−5.637	−4.476	−5.712	−3.238
Acenaphthene	ACEN	−4.821	−5.788	−3.013	−5.788	−4.511	−5.747	−3.273
Fluorene	FLUO	−4.756	−5.427	−2.667	−5.427	−4.163	−5.395	−2.930
Phenanthrene	PHE	−4.500	−5.015	−2.269	−5.015	−3.724	−4.953	−2.493
Anthracene	ANT	−3.811	−5.099	−2.351	−5.099	−3.459	−4.687	−2.231
2-Methylphenanthrene	2-MPHE	−3.747	−4.832	−2.091	−4.832	−3.205	−4.430	−1.980
3,6-Dimethylphenanthrene	3,6-DMPHE	−3.847	−4.488	−1.752	−4.488	−2.728	−3.951	−1.504
Fluoranthene	FLUA	−3.223	−4.121	−1.387	−4.121	−2.946	−4.172	−1.722
Pyrene	PYR[b]	−3.027	−4.385	−1.650	−4.385	−2.950	−4.179	−1.723
Retene	RET	−2.703	−4.056	−1.322	−4.056	−1.919	−3.149	−0.689
Benzo[a]anthracene	BaA[b]	−1.592	−3.819	−1.084	−3.819	−1.828	−3.065	−0.591
Benzo[e]pyrene	BeP	−0.316	−2.375	0.406	−2.375	−1.513	−2.756	−0.270
Benzo[a]pyrene	BaP	0.028	−2.683	0.083	−2.683	−1.016	−2.271	0.238
Indeno [1,2,3-cd]pyrene	IcdP	0.255	−2.250	0.538	−2.250	−0.284	−1.557	0.991
Dibenzo[a,h]anthracene	DahA	−0.687	−2.106	0.691	−2.106	−0.094	−1.377	1.191
Benzo[g,h,i]perylene	BghiP	0.028	−2.281	0.505	−2.281	−0.702	−1.967	0.562
1-Indanone	1-IND	−3.998	−5.934	−3.151	−5.934	−4.235	−5.490	−2.981

Compound	Abbreviations	Exp. log K_P	Log K_{OA} model			MLR prediction		
			Pred. log K_P	LLCI	ULCI	Pred. log K_P	LLCI	ULCI
1,4-Naphthoquinone	1,4-NQ	−3.990	−3.992	−1.258	−3.992	−4.261	−5.496	−3.029
1-Naphthaldehyde	1-NALD[b]	−4.111	−5.054	−2.307	−5.054	−3.506	−4.738	−2.272
2-Biphenylcarboxaldehyde	2-BPCA[b]	−3.491	−4.605	−1.867	−4.605	−2.760	−3.990	−1.531
9-Fluorenone	9-FLU	−3.630	−4.417	−1.682	−4.417	−2.959	−4.183	−1.737
1,2-Acenaphthenequinone	1,2-ACEQ	−3.196	−3.992	−1.258	−3.992	−3.180	−4.409	−1.950
9,10-Anthraquinone	9,10-AQ[b]	−2.382	−3.602	−0.864	−3.602	−2.902	−4.116	−1.687
1,8-Naphtalic anhydride	1,8-NA[b]	−3.033	−4.611	−1.874	−4.611	−3.140	−4.367	−1.913
4H-Cyclopenta[d,e,f]phenanthrenone	4-CPHE[b]	−2.739	−3.480	−0.740	−3.480	−2.345	−3.564	−1.124
2-Meth-9,10-anthraquinone	2-MAQ	−1.944	−2.764	−0.002	−2.764	−2.362	−3.579	−1.147
Benzo[a]florenone	BAFLU[b]	−1.590	−3.036	−0.284	−3.036	−1.322	−2.552	−0.092
7H-Benzo[d,e]anthracene-7-one	BdeAQ[b]	−0.682	−2.985	−0.232	−2.985	−1.328	−2.563	−0.097
Benzo[a]anthracene-7,12-dione	BaAQ	−1.112	−1.783	1.036	−1.783	−1.231	−2.457	−0.003
5,12-Naphthacenequinone	5,12-NQ	−0.949	−1.708	1.116	−1.708	−1.006	−2.238	0.226
6H-Benzo[c,d]pyren-6-one	BcdPQ[b]	−0.635	−2.100	0.697	−2.100	−0.592	−1.841	0.656
1-Nitronaphthalene	1-NNAP	−3.703	−4.943	−2.199	−4.943	−3.635	−4.862	−2.409
2-Nitrobiphenyl	2-NBP	−2.352	−4.670	−1.931	−4.670	−3.184	−4.400	−1.969
5-Nitroacenaphthene	5-NACE	−2.219	−4.385	−1.650	−4.385	−2.867	−4.091	−1.644
2-Nitrofluorene	2-NFLU	−1.932	−4.547	−1.810	−4.547	−2.501	−3.719	−1.283
9-Nitrophenanthrene	9-NPHE	−2.098	−3.710	−0.974	−3.710	−2.324	−3.539	−1.108
9-Nitroanthracene	9-NANT	−1.858	−3.315	−0.571	−3.315	−1.660	−2.889	−0.434
1-Nitropyrene	1-NPYR	−1.496	−2.639	0.129	−2.639	−1.048	−2.285	0.186
2,7-Dinitrofluorene	2,7-DNFLU	−1.595	−3.023	−0.271	−3.023	−2.037	−3.254	−0.821
6-Nitrochrysene	6-NCHR	−1.696	−2.325	0.459	−2.325	−0.604	−1.845	0.638
Quinoline	QUI	−3.127	−5.683	−2.913	−5.683	−3.731	−4.994	−2.466

Compound	Abbreviations	Exp. log K_P	Log K_{OA} model			MLR prediction		
			Pred. log K_P	LLCI	ULCI	Pred. log K_P	LLCI	ULCI
Benzo[h]quinoline	BhQ[b]	−2.804	−4.134	−1.400	−4.134	−2.483	−3.716	−1.252
Acridine	ACR	−2.275	−3.889	−1.155	−3.889	−1.969	−3.219	−0.721
Carbazole	CAR[b]	−3.372	−3.838	−1.103	−3.838	−4.071	−5.301	−2.843

LLCI: lower limit of 95% confidence interval; ULCI: upper limit of 95% confidence interval
